# *Francisella tularensis* subsp. *tularensis* Group A.I, United States

**DOI:** 10.3201/eid2005.131559

**Published:** 2014-05

**Authors:** Dawn N. Birdsell, Anders Johansson, Caroline Öhrman, Emily Kaufman, Claudia Molins, Talima Pearson, Miklós Gyuranecz, Amber Naumann, Amy J. Vogler, Kerstin Myrtennäs, Pär Larsson, Mats Forsman, Andreas Sjödin, John D. Gillece, James Schupp, Jeannine M. Petersen, Paul Keim, David M. Wagner

**Affiliations:** Northern Arizona University, Flagstaff, Arizona, USA (D.N. Birdsell, E. Kaufman, T. Pearson, A. Naumann, A.J. Vogler, P. Keim, D.M. Wagner);; Umeå University, Umeå, Sweden (A. Johansson);; Swedish Defense Research Agency, Umeå (C. Öhrman, K. Myrtennäs, P. Larsson, M. Forsman, A. Sjödin);; Centers for Disease Control and Prevention, Fort Collins, Colorado, USA (C. Molins, J.M. Petersen);; Center for Agricultural Research, Hungarian Academy of Sciences, Budapest, Hungary (M. Gyuranecz);; Translational Genomics Research Institute, Flagstaff (J. Gillece, J. Schupp, P. Keim)

**Keywords:** Francisella tularensis subsp. tularensis, Francisella tularensis, phylogeography, SNP, single-nucleotide polymorphism, subgroup, United States, group A.I, bacteria, geographic distribution, tularemia

## Abstract

We used whole-genome analysis and subsequent characterization of geographically diverse strains using new genetic signatures to identify distinct subgroups within *Francisella tularensis* subsp. *tularensis* group A.I: A.I.3, A.I.8, and A.I.12. These subgroups exhibit complex phylogeographic patterns within North America. The widest distribution was observed for A.I.12, which suggests an adaptive advantage.

Tularemia, caused by the bacterium *Francisella tularensis*, is a potentially severe disease that often causes unspecific symptoms; because of its low infectious dose and ease of dissemination, *F. tularensis* is considered a category A biothreat agent (*1*). Three subspecies of *F. tularensis* have been identified; *F. tularensis* subsp. *tularensis* (type A) has been identified only in North America. Numerous subtyping schemes have subdivided type A into 2 groups, A.I and A.II (*2*–*8*). Group A.II is found primarily in the western United States (*3*,*4*), whereas group A.I is found throughout the central and eastern regions of the country and sporadically in some western states (*3*,*4*,*9*).

Groups A.I and A.II differ in virulence, as do subgroups within A.I, although clinical signs and symptoms can be similar. Human infections involving A.I strains are associated with a higher fatality rate than that for infections involving A.II strains (*4*,*10*); this finding was experimentally confirmed in mice (*11*). Kugeler et al. (*10*) used pulsed-field gel electrophoresis (PFGE) to identify 2 subgroups within A.I, A1a and A1b; this study found A1b strains were associated with higher death rates and were more often isolated from human tissue types that were associated with severe disease. This difference was also experimentally confirmed in mice (*11*,*12*). However, virulence testing is not often used in clinical settings because it is slow, complicated, and expensive. Thus, molecular approaches that can rapidly assign an unknown strain to one of the recognized groups with known differences in virulence may provide valuable information to clinicians.

Because PFGE lacks the phylogenetic resolution of some other testing methods (*6*), we independently identified genetic subgroups within A.I by conducting whole-genome sequencing (WGS) of 13 A.I strains (Figure 1; Table 1, Appendix). The 13 strains were selected on the basis of assignment to PFGE subgroups A1a or A1b (*10*) and to maximize geographic diversity; the previously sequenced A.I strain Schu S4 (*13*) was also included. WGS data were generated, assembled, and analyzed as described in the Technical Appendix (wwwnc.cdc.gov/EID/article/20/5/13-1559-Techapp1.pdf).

**Figure 1:**
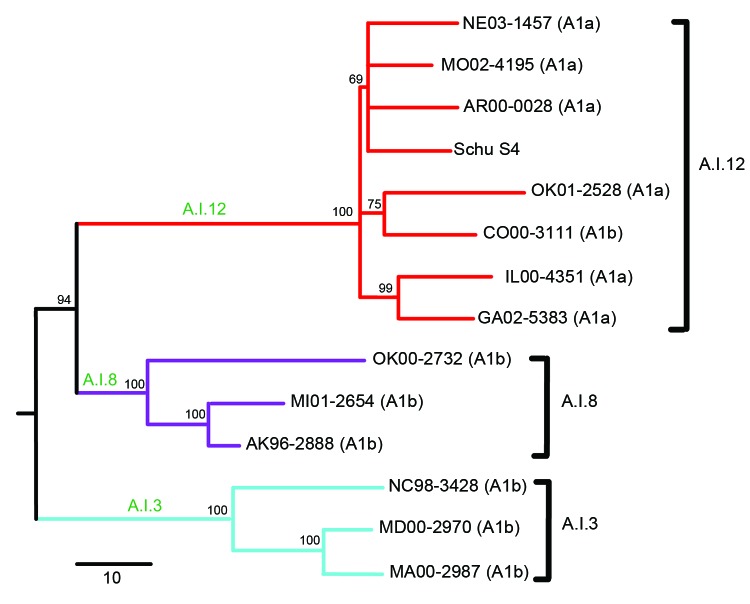
Neighbor-joining tree of 14 *Francisella tularensis* subsp. *tularensis* group A.I strains constructed on the basis of single-nucleotide polymorphisms (SNPs) discovered from whole-genome sequencing. Lines represent major groups within A.I: red, A.I.12; purple, A.I.8; blue, A.I.3. Branch nomenclature for each group is indicated by green text. Bootstrap values for each group and subpopulation are indicated in black font. Pulsed-field gel electrophoresis classifications (A1a and A1b) are indicated for each sequenced strain. A.I strain SchuS4 (GenBank accession no. NC_006570) was included as a reference strain. Scale bar indicates no. SNPs.

**Table 1:** Strains of *Francisella tularensis* subsp. *tularensis* used in study of geographic distribution of group A.I strains, United States*

Strain ID†	Original strain ID‡	Original laboratory	State/province of exposure	Region§	Year	CDC PFGE type¶	Subclade#	Major group	Subgroup
AR01-0903	AR01-0903	CDC	AR	C	2001	A1	A.I.1/2	A.I.12	A.I.12/2
AR01-1117	AR01-1117	CDC	AR	C	2001	A1b	A.I.1/2	A.I.8	A.I.9
AR03-1002	AR03-1002	CDC	AR	C	2003	A1a	A.I.SchuS4	A.I.12	A.I.16
AR97-0782	AR97-0782	CDC	AR	C	1997	A1a	A.I.SchuS4	A.I.12	A.I.16
AR98-2146	AR98-2146	CDC	AR	C	1998	A1a	A.I.SchuS4	A.I.12	A.I.16
AR99-3840	AR99-3840	CDC	AR	C	1999	A1b	A.I.1/2	A.I.8	A.I.9
CA04-2148	CA04-2148	CDC	CA	W	1983	A1b	A.I.1/2	A.I.8	A.I.9
CA04-2149	CA04-2149	CDC	CA	W	2004	A1a	A.I.SchuS4	A.I.12	A.I.14
CA04-2258	CA04-2258	CDC	CA	W	2004	A1b	A.I.1/2	A.I.8	A.I.9
CA95-1135	CA95-1135	CDC	CA	W	1995	A1	A.I.1/2	A.I.8	A.I.9
DE03-1544	DE03-1544	CDC	DE	E	2003	A1b	A.I.1/2	A.I.3	A.I.4
F0003	FSC 043	FOI	OH	E	1941	NA	A.I.SchuS4	A.I.12	A.I.13
F0005	FSC 041	FOI	BC	W	1935	NA	A.I.1/2	A.I.8	A.I.9
F0008	FSC 046	FOI	OH	E	1940	NA	A.I.SchuS4	A.I.12	A.I.13
F0061	KS00-0948	CDC	KS	C	2000	A1a	A.I.SchuS4	A.I.12	A.I.16
F0069	OK00-2731	CDC	OK	C	2000	A1b	A.I.SchuS4	A.I.12	A.I.14
F0071	OK00-2733	CDC	OK	C	2000	A1a	A.I.SchuS4	A.I.12	A.I.13
F0076	OK-ADA	OKDH	OK	C	2000	NA	A.I.SchuS4	A.I.12	A.I.13
F0077	OK-CAN	OKDH	OK	C	1993	NA	A.I.SchuS4	A.I.12	A.I.13
F0078	OK-CHK	OKDH	OK	C	2000	NA	A.I.SchuS4	A.I.12	A.I.14
F0079	OK-HUG	OKDH	OK	C	1993	NA	A.I.SchuS4	A.I.12	A.I.13
F0080	OK-OKL-1	OKDH	OK	C	1994	NA	A.I.SchuS4	A.I.12	A.I.2/13/14/15
F0084	OK-TUL-2	OKDH	OK	C	1996	NA	A.I.SchuS4	A.I.12	A.I.14
F0085	OK-TUL-3	OKDH	OK	C	1993	NA	A.I.1/2	A.I.8	A.I.11
F0244	83A-5698	CADH	CA	W	1983	NA	A.I.1/2	A.I.8	A.I.9
F0246	93A-5254	CADH	CA	W	1993	NA	A.I.1/2	A.I.8	A.I.9
F0272	92A-4575	CADH	CA	W	1992	NA	A.I.1/2	A.I.8	A.I.9
F0276	94A-3325	CADH	CA	W	1994	NA	A.I.1/2	A.I.8	A.I.9
F0281	MO01-2148	CDC	MO	C	2001	A1a	A.I.SchuS4	A.I.12	A.I.13
F0282	KS00-1817	CDC	KS	C	2000	A1a	A.I.1/2	A.I.12	A.I.12/2
F0287	AR99-3448	CDC	AR	C	1999	A1b	A.I.SchuS4	A.I.12	A.I.14
F0297	FSC 047	FOI	AK	W	UNK	NA	A.I.1/2	A.I.8	A.I.9
F0298	FSC 052	FOI	AK	W	UNK	NA	A.I.1/2	A.I.8	A.I.9
F0307	ND00-0952	CDC	ND	C	2000	A1a	A.I.1/2	A.I.12	A.I.12/2
F0308	MA01-2505	CDC	MA	E	1978	NA	A.I.4	A.I.3	A.I.4
F0312	CO99-1817	CDC	CO	W	1999	A1	A.I.1/2	A.I.12	A.I.12/2
F0314	MO01-4055	CDC	MO	C	2001	A1b	A.I.1/2	A.I.8	A.I.9
F0315	MO01-1907	CDC	MO	C	2001	A1a	A.I.SchuS4	A.I.12	A.I.16
F0332	NY98-1732	CDC	NY	E	1998	A1b	A.I.4	A.I.3	A.I.4
F0347	GA02-5426	CDC	MA	E	1981	A1b	A.I.SchuS4	A.I.12	A.I.14
F0348	AR03-1800	CDC	AR	C	2003	A1b	A.I.1/2	A.I.8	A.I.9
F0357	SD97-2107	CDC	SD	C	1997	A1b	A.I.SchuS4	A.I.12	A.I.14
F0359	NC01-5379	CDC	NC	E	2001	A1b	A.I.4	A.I.3	A.I.4
F0368	KS03-2487	CDC	KS	C	2003	A1b	A.I.1/2	A.I.8	A.I.9
F0375	MO02-1911	CDC	MO	C	2002	A1a	A.I.SchuS4	A.I.12	A.I.13
F0379	PA03-3835	CDC	PA	E	2003	A1a	A.I.SchuS4	A.I.12	A.I.13
F0383	OSU-1	OSU	OK	C	UNK	NA	A.I.SchuS4	A.I.12	A.I.14
F0384	OSU-2	OSU	OK	C	UNK	NA	A.I.1/2	A.I.12	A.I.12/2
F0385	OSU-3	OSU	OK	C	UNK	NA	A.I.1/2	A.I.8	A.I.11
F0386	OSU-4	OSU	OK	C	UNK	NA	A.I.SchuS4	A.I.12	A.I.14
F0387	OSU-5	OSU	OK	C	UNK	NA	A.I.SchuS4	A.I.12	A.I.14
F0388	OSU-6	OSU	OK	C	UNK	NA	A.I.SchuS4	A.I.12	A.I.16
F0389	OSU-7	OSU	OK	C	UNK	NA	A.I.1/2	A.I.8	A.I.11
F0390	OSU-8	OSU	OK	C	UNK	NA	A.I.SchuS4	A.I.12	A.I.13
F0391	OSU-9	OSU	OK	C	UNK	NA	A.I.SchuS4	A.I.12	A.I.14
F0392	OSU-10	OSU	OK	C	UNK	NA	A.I.1/2	A.I.8	A.I.9
F0393	OSU-11	OSU	OK	C	UNK	NA	A.I.SchuS4	A.I.12	A.I.14
F0394	OSU-12	OSU	OK	C	UNK	NA	A.I.SchuS4	A.I.12	A.I.16
F0395	OSU-13	OSU	OK	C	UNK	NA	A.I.SchuS4	A.I.12	A.I.13
F0396	OSU-14	OSU	OK	C	UNK	NA	A.I.SchuS4	A.I.12	A.I.14
F0397	OSU-15	OSU	OK	C	UNK	NA	A.I.SchuS4	A.I.12	A.I.13
F0398	OSU-16	OSU	OK	C	UNK	NA	A.I.SchuS4	A.I.12	A.I.13
F0399	OSU-17	OSU	OK	C	UNK	NA	A.I.SchuS4	A.I.12	A.I.14
F0401	OSU-19	OSU	OK	C	UNK	NA	A.I.SchuS4	A.I.12	A.I.13
F0402	OSU-20	OSU	OK	C	UNK	NA	A.I.1/2	A.I.8	A.I.9
F0403	OSU-21	OSU	OK	C	UNK	NA	A.I.1/2	A.I.12	A.I.12/2
F0404	OKH-1	OSU/OKDH	OK	C	UNK	NA	A.I.SchuS4	A.I.12	A.I.13
F0405	OKH-2	OSU/OKDH	OK	C	UNK	NA	A.I.SchuS4	A.I.12	A.I.2/13/14/15
F0406	OKH-3	OSU/OKDH	OK	C	UNK	NA	A.I.SchuS4	A.I.12	A.I.14
F0407	OKH-4	OSU/OKDH	OK	C	UNK	NA	A.I.SchuS4	A.I.12	A.I.14
F0408	OKH-5	OSU/OKDH	OK	C	UNK	NA	A.I.SchuS4	A.I.12	A.I.13
F0409	OKH-6	OSU/OKDH	OK	C	UNK	NA	A.I.SchuS4	A.I.12	A.I.13
F0410	OKH-7	OSU/OKDH	OK	C	UNK	NA	A.I.SchuS4	A.I.12	A.I.13
F0411	OKH-8	OSU/OKDH	OK	C	UNK	NA	A.I.SchuS4	A.I.12	A.I.14
F0412	OKH-9	OSU/OKDH	OK	C	UNK	NA	A.I.SchuS4	A.I.12	A.I.14
F0413	OKH-10	OSU/OKDH	OK	C	UNK	NA	A.I.1/2	A.I.8	A.I.11
F0414	NC-1	OSU	NC	E	1998	NA	A.I.4	A.I.3	A.I.4
F0415	NC-2	OSU	NC	E	1998	NA	A.I.4	A.I.3	A.I.4
F0685	80700069	LLNL	UT	W	2007	NA	A.I.1/2	A.I.12	A.I.12/2
F0690	1100558	IAB	AK	W	2003	NA	A.I.1/2	A.I.8	A.I.9
F0691	1133496	IAB	AK	W	2004	NA	A.I.1/2	A.I.8	A.I.9
F0693	1211988	IAB	AK	W	2006	NA	A.I.1/2	A.I.8	A.I.8/9/10
F0694	1211990	IAB	AK	W	2006	NA	A.I.1/2	A.I.8	A.I.8/9/10
F0695	1213860	IAB	AK	W	2006	NA	A.I.1/2	A.I.8	A.I.9
F0696	1213861	IAB	AK	W	2006	NA	A.I.1/2	A.I.8	A.I.9
F0697	0916900084	IAB	AK	W	2009	NA	A.I.1/2	A.I.8	A.I.9
F0717**††	OK01-2528	CDC	OK	C	2001	A1a	A.I.SchuS4	A.I.12	A.I.14
F0718**††	MO02-4195	CDC	MO	C	2002	A1a	A.I.SchuS4	A.I.12	A.I.13
F0723**‡‡	MD00-2970	CDC	DE	E	2000	A1b	A.I.4	A.I.3	A.I.4
F0724**‡‡	MA00-2987	CDC	MA	E	2000	A1b	A.I.4	A.I.3	A.I.4
GA00-3376	GA00-3376	CDC	GA	E	2000	A1a	A.I.SchuS4	A.I.12	A.I.2/13/14/15
GA02-5347	GA02-5347	CDC	MS	C	1980	A1a	A.I.SchuS4	A.I.12	A.I.16
GA02-5351	GA02-5351	CDC	LA	C	1981	A1a	A.I.SchuS4	A.I.12	A.I.13
GA02-5353	GA02-5353	CDC	TN	E	1992	A1a	A.I.SchuS4	A.I.12	A.I.16
GA02-5360	GA02-5360	CDC	KY	E	1973	A1b	A.I.SchuS4	A.I.12	A.I.14
GA02-5373	GA02-5373	CDC	NY	E	1977	A1b	A.I.1/2	A.I.3	A.I.4
GA02-5381	GA02-5381	CDC	AR	C	1979	A1b	A.I.1/2	A.I.8	A.I.10/11
GA02-5386	GA02-5386	CDC	OR	W	1978	A1a	A.I.SchuS4	A.I.12	A.I.13
GA02-5388	GA02-5388	CDC	SD	C	1985	A1b	A.I.1/2	A.I.8	A.I.9
GA02-5389	GA02-5389	CDC	IA	C	1979	A1a	A.I.SchuS4	A.I.12	A.I.13
GA02-5390	GA02-5390	CDC	MS	C	1981	A1a	A.I.SchuS4	A.I.12	A.I.13
GA02-5392	GA02-5392	CDC	LA	C	1983	A1a	A.I.SchuS4	A.I.12	A.I.15/16
GA02-5393	GA02-5393	CDC	TN	E	1980	A1a	A.I.1/2	A.I.12	A.I.12/2
GA02-5395	GA02-5395	CDC	NE	C	1982	A1a	A.I.SchuS4	A.I.12	A.I.13
GA02-5403	GA02-5403	CDC	OR	W	1984	A1b	A.I.1/2	A.I.8	A.I.11
GA02-5404	GA02-5404	CDC	GA	E	1986	A1b	A.I.SchuS4	A.I.3	A.I.5/6
GA02-5406	GA02-5406	CDC	DE	E	1987	A1a	A.I.SchuS4	A.I.12	A.I.2/13/14/15
GA02-5407	GA02-5407	CDC	AR	C	1980	A1b	A.I.SchuS4	A.I.12	A.I.14
GA02-5408	GA02-5408	CDC	SD	C	1983	A1b	A.I.SchuS4	A.I.12	A.I.12/2
GA02-5409	GA02-5409	CDC	KS	C	1980	A1b	A.I.1/2	A.I.3	A.I.5/6
GA02-5410	GA02-5410	CDC	SD	C	1984	A1a	A.I.SchuS4	A.I.12	A.I.14
GA02-5412	GA02-5412	CDC	TX	C	1980	A1a	A.I.SchuS4	A.I.12	A.I.16
GA02-5422	GA02-5422	CDC	TX	C	1983	A1a	A.I.SchuS4	A.I.12	A.I.16
GA02-5425	GA02-5425	CDC	GA	E	1986	A1a	A.I.SchuS4	A.I.12	A.I.16
GA02-5427	GA02-5427	CDC	OH	E	1986	A1a	A.I.SchuS4	A.I.12	A.I.16
GA02-5428	GA02-5428	CDC	MO	C	1980	A1b	A.I.1/2	A.I.8	A.I.11
GA02-5435	GA02-5435	CDC	LA	C	1984	A1b	A.I.1/2	A.I.8	A.I.10/11
GA02-5441	GA02-5441	CDC	LA	C	1983	A1b	A.I.SchuS4	A.I.12	A.I.14
GA02-5444	GA02-5444	CDC	TX	C	1980	A1a	A.I.SchuS4	A.I.12	A.I.14
GA02-5445	GA02-5445	CDC	TN	E	1992	A1a	A.I.SchuS4	A.I.12	A.I.2/13/14/15
GA02-5446	GA02-5446	CDC	NC	E	1992	A1b	A.I.1/2	A.I.3	A.I.5/6
GA02-5449	GA02-5449	CDC	TN	E	1989	A1a	A.I.1/2	A.I.12	A.I.16
GA02-5454	GA02-5454	CDC	MO	C	1987	A1b	A.I.1/2	A.I.8	A.I.10/11
GA02-5462	GA02-5462	CDC	OK	C	1984	A1a	A.I.SchuS4	A.I.12	A.I.16
GA02-5465	GA02-5465	CDC	IL	C	1985	A1a	A.I.SchuS4	A.I.12	A.I.14
GA02-5469	GA02-5469	CDC	OK	C	1981	A1b	A.I.1/2	A.I.8	A.I.11
GA02-5470	GA02-5470	CDC	MO	C	1981	A1b	A.I.SchuS4	A.I.12	A.I.14
GA02-5476	GA02-5476	CDC	KS	C	1982	A1b	A.I.SchuS4	A.I.12	A.I.14
GA02-5482	GA02-5482	CDC	IN	E	1981	A1a	A.I.SchuS4	A.I.12	A.I.16
GA02-5497	GA02-5497	CDC	VA	E	1982	A1a	A.I.1/2	A.I.12	A.I.12/2
GA02-5499	GA02-5499	CDC	MD	E	1982	A1b	A.I.SchuS4	A.I.3	A.I.4
GA02-5500	GA02-5500	CDC	MO	C	1982	A1a	A.I.SchuS4	A.I.12	A.I.13
GA02-5501	GA02-5501	CDC	LA	C	1982	A1b	A.I.SchuS4	A.I.12	A.I.14
GA02-5512	GA02-5512	CDC	MA	E	1981	A1a	A.I.SchuS4	A.I.12	A.I.16
GA99-2584	GA99-2584	CDC	GA	E	1999	A1b	A.I.1/2	A.I.3	A.I.5/6
F0719**	AR00-0028	CDC	AR	C	2000	A1a	A.I.SchuS4	A.I.12	A.I.13
F0720**	NE03-1457	CDC	NE	C	2003	A1a	A.I.SchuS4	A.I.12	A.I.13
F0721**	IL00-4351	CDC	IL	C	2000	A1a	A.I.SchuS4	A.I.12	A.I.16
F0722**	GA02-5383	CDC	GA	E	2002	A1a	A.I.SchuS4	A.I.12	A.I.16
F0725**	MI01-2654	CDC	MI	E	2001	A1b	A.I.1/2	A.I.8	A.I.9
F0726**	AK96-2888	CDC	AK	W	1996	A1b	A.I.1/2	A.I.8	A.I.9
F0727**	OK00-2732	CDC	OK	C	2000	A1b	A.I.1/2	A.I.8	A.I.11
F0728**	CO00-3111	CDC	CO	W	2000	A1b	A.I.SchuS4	A.I.12	A.I.14
F0729**	NC98-3428	CDC	NC	E	1998	A1b	A.I.1/2	A.I.3	A.I.6
IA00-3490	IA00-3490	CDC	IA	C	2000	A1a	A.I.SchuS4	A.I.12	A.I.13
ID04-2686	ID04-2686	CDC	ID	W	2004	A1b	A.I.1/2	Basal	A.I.1/3/7
IL01-3022	IL01-3022	CDC	IL	C	2001	A1a	A.I.SchuS4	A.I.12	A.I.16
KS01-4799	KS01-4799	CDC	KS	C	2001	A1b	A.I.1/2	A.I.8	A.I.9
KS03-2455	KS03-2455	CDC	KS	C	2003	A1a	A.I.SchuS4	A.I.12	A.I.13
KS82-0004	KS82-0004	CDC	KS	C	1982	A1a	A.I.SchuS4	A.I.12	A.I.14
KS89-0548	KS89-0548	CDC	KS	C	1989	A1b	A.I.SchuS4	A.I.12	A.I.14
KY00-2794	KY00-2794	CDC	KY	E	2000	A1a	A.I.SchuS4	A.I.12	A.I.13
LA95-0751	LA95-0751	CDC	LA	C	1995	A1	A.I.SchuS4	A.I.12	A.I.14
MA04-2790	MA04-2790	CDC	MA	E	2004	A1b	A.I.1/2	A.I.3	A.I.4
MD01-1249	MD01-1249	CDC	MD	E	2001	A1b	A.I.1/2	A.I.3	A.I.5/6
MD04-2528	MD04-2528	CDC	MD	E	2004	A1b	A.I.1/2	A.I.3	A.I.4
NC05-1521	NC05-1521	CDC	NC	E	2005	A1b	A.I.1/2	A.I.3	A.I.5/6
NC98-3687	NC98-3687	CDC	NC	E	1998	A1	A.I.1/2	A.I.3	A.I.4
ND00-1213	ND00-1213	CDC	ND	C	2000	A1a	A.I.SchuS4	A.I.12	A.I.16
ND01-1900	ND01-1900	CDC	ND	C	2001	A1a	A.I.1/2	Basal	A.I.1/3/7
NE82-0001	NB82-0001	CDC	NE	C	1982	A1b	A.I.1/2	A.I.3	A.I.5/6
NE88-5553	NB88-5553	CDC	NE	C	1988	A1b	A.I.1/2	A.I.8	A.I.10/11
NE95-1835	NE95-1835	CDC	NE	C	1995	A1a	A.I.1/2	A.I.12	A.I.12/2
NE97-2086	NE97-2086	CDC	NE	C	1997	A1a	A.I.SchuS4	A.I.12	A.I.14
NJ04-3009	NJ04-3009	CDC	NJ	E	2004	A1b	A.I.1/2	A.I.3	A.I.4
NY04-2564	NY04-2564	CDC	NY	E	2004	A1b	A.I.1/2	A.I.3	A.I.4
NY96-3369	NY96-3369	CDC	NY	E	1996	A1b	A.I.1/2	A.I.3	A.I.4
OK82-0005	OK82-0005	CDC	OK	C	1982	A1a	A.I.SchuS4	A.I.12	A.I.13
PA84-0001	PA84-0001	CDC	PA	E	1984	A1a	A.I.SchuS4	A.I.12	A.I.13
SD00-3147	SD00-3147	CDC	SD	C	2000	A1a	A.I.SchuS4	A.I.12	A.I.14
SD04-0857	SD04-0857	CDC	SD	C	2003	A1a	A.I.SchuS4	A.I.12	A.I.14
TX05-5417	TX05-5417	CDC	TX	C	2005	A1b	A.I.1/2	Basal	A.I.1/3/7
UT07-4262	UT07-4262	CDC	UT	W	2007	A1a	A.I.1/2	A.I.12	A.I.12/2
UT07-4263	UT07-4263	CDC	UT	W	2007	A1a	A.I.1/2	A.I.12	A.I.12/2
UT98-3134	UT98-3134	CDC	UT	W	1998	A1a	A.I.SchuS4	A.I.12	A.I.14
VA00-1000	VA00-1000	CDC	VA	E	2000	A1b	A.I.1/2	A.I.3	A.I.5/6
VA00-2108	VA00-2108	CDC	VA	E	2000	A1	A.I.1/2	A.I.3	A.I.4
VA98-5912	VA98-5912	CDC	VA	E	1998	A1b	A.I.1/2	A.I.3	A.I.3/4/5
VA99-2600	VA99-2600	CDC	VA	E	1999	A1b	A.I.1/2	A.I.3	A.I.4

*ID, identification; CDC, Centers for Disease Control and Prevention (Fort Collins, CO, USA); PFGE, pulsed-field gel electrophoresis; C, central; W, western; E, eastern; FOI, Swedish Defence Research Agency (Umeå, Sweden); NA, not applicable; BC, British Columbia, Canada; OKDH, Oklahoma State Department of Health (Oklahoma City, OK, USA); CADH, California Department of Health Services (Richmond, CA, USA); OSU, Oklahoma State University (Stillwater, OK, USA); LLNL, Lawrence Livermore National Laboratory (Livermore, CA, USA); IAB, Institute of Arctic Biology University of Alaska (Fairbanks, AK, USA). †Strain ID from the Northern Arizona University (Flagstaff, AZ, USA) or CDC collections. ‡Strain ID from the originating laboratory. §Region classification of state/province for genetic diversity calculation. ¶See (*10*). #Canonical single nucleotide polymorphism subgroup as originally defined in (*7**,**8*). Names used here have been modified to conform to the nomenclature standard established in (*14*). **Whole genome sequenced in this study. ††Mouse study; lower-virulence A.I strain (*11*). ‡‡Mouse study; higher-virulence A.I strain (*11*).

Our whole-genome phylogeny revealed 3 major subgroups within *F. tularensis* subsp. *tularensis* A.I: A.I.3, A.I.8, and A.I.12 (Figure 1). The names we assigned to these subgroups are consistent with previous phylogenetic nomenclature within *F. tularensis* (*14*). With the exception of 1 strain (ND01-1900) that was not assigned to any of the 3 subgroups, all strains previously assigned to PFGE subgroup A1a belonged to the newly designated A.I.12 subgroup (Figure 1; Table 1). In contrast, strains previously assigned to PFGE subgroup A1b were distributed among all 3 of the new subgroups (Figure 1; Table 1). We concluded that results of characterization of subgroups A1a and A1b by PFGE are not in agreement with findings of a robust whole-genome phylogeny and therefore focused the remainder of our analysis on subgroups identified by using WGS.

We observed several differences among the 3 subgroups in the whole-genome phylogeny (Figure 1). The first split separated the A.I.3 subgroup from the A.I.8 and A.I.12 subgroups; a second split separated the A.I.8 and A.I.12 subgroups. A long branch of 25 single nucleotide polymorphisms (SNPs) led to the A.I.3 subgroup, in which relatedness among the sequenced strains was moderate. A branch of 9 SNPs led to the A.I.8 subgroup, and again, relatedness among the sequenced strains was moderate. The branch leading to subgroup A.I.12 was, by comparison, much longer (37 SNPs), and the sequenced strains were separated only by 3 short branches (1–4 SNPs). This pattern of several short branches without hierarchical structuring is consistent with a recent radiation, an evolutionary process in response to adaptive change, new ecologic opportunities, or a combination of these factors.

To show more comprehensive phylogenetic patterns, we developed 16 canonical SNP (canSNP) assays as described (Technical Appendix) and used them to screen 179 *F. tularensis* subsp. *tularensis* A.I strains selected from the collections of the Centers for Disease Control and Prevention (Fort Collins, CO, USA). We selected strains that were representative of all states where A.I infections occur and of all PFGE classification types (Table 1). One limitation of our study is that we did not analyze an equal number of strains from all regions of the country. However, our sample reflects the distribution of human disease caused by *F. tularensis* subsp. *tularensis* A.I strains: prevalent in the central United States, less common in the eastern United States, and rare in the western United States (*4*). The canSNP assays were based on 12 SNP signatures (Table 2) from the whole-genome phylogeny (Figure 1) and 4 previously described SNP signatures (*6*–*8*). Using these assays, we assigned the 179 strains to 15 *F. tularensis* subsp. *tularensis* A.I subpopulations, including 8 intervening nodes (Figure 2, panel A). We found 6 subpopulations in the A.I.12 subgroup, 4 in A.I.8, and 4 in A.I.3 (Table 1). To identify broad phylogeographic patterns, we created maps indicating specific states where strains from the 15 subpopulations were isolated (Figure 2, panel B). Within these maps, we created boundaries corresponding to 3 regions within the United States: western, central, and eastern.

**Table 2:** Melt-MAMA primers targeting canSNPs for new phylogenetic branches in *Francisella tularensis* subsp. *tularensis* A.I in United States*

		SchuS4† position	SNP state, der/anc‡	Primers, 5′ → 3′§	Con¶	Temp, °C#
Subgroup						
Major	Minor					
NA	A.I.7	1005448**	C/T	A: TATTTCAATTTTTGCGATGGTAgGT	0.80	55
				D: ggggcggggcggggcTATTTCAATTTTTGCGATGGTAcTC	0.20	
				C: AAGTATGTTGGCAAGTAAAGTGAGAAGA	0.20	
A.I.12	NA	142781††	C/G	A: GCTTATCGCCGACATTCATCtAC	0.20	60
				D: ggggcggggcggggcgggCTTATCGCCGACATTCATCcAG	0.20	
				C: GGTATGGCAAAAAATACTTATGGTACG	0.20	
A.I.12	A.I.13	1833651‡‡	T/C	A: CTTTCAATCATGTAACCATCATTATTTAaGC	0.80	60
				D: cggggcggggcggggcggggCTTTCAATCATGTAACCATCATTATTTAgGT	0.20	
				C: CTTAATGAACTTGGTGTAATGGGTAGATA	0.20	
A.I.12	A.I.16	273622	T/C	A: AAACTTAAAAAAGAGCAAGAACTTAATGATcTC	0.60	60
				D: ggggcggggcggggcgAAACTTAAAAAAGAGCAAGAACTTAATGATaTT	0.15	
				C: CATCTTCATTAAAAGTCTTATTGTTTAAACGC	0.15	
A.I.12	A.I.15	1210286	A/G	A: TCTTAAAACATCGACACTCTCAACcTG	0.80	60
				D: ggggcggggcggggcGATCTTAAAACATCGACACTCTCAACtTA	0.20	
				C: gtatcattcagatcataatgaagcaactatc	0.20	
A.I.12	A.I.14	1296147	T/C	A: ATCATACTGGTTATATTGGCGGTcTC	0.80	60
				D: cggggcggggcggggcggggATCATACTGGTTATATTGGCGGTgTT	0.20	
				C: GATGAGTCGCTATTAGCTTCTCGAAAG	0.20	
A.I.8	NA	1150298	G/A	A: TAGTCAATCTTGGAACTCCAGAtAA	0.75	60
				D: ggggcggggcggggcTAGTCAATCTTGGAACTCCAGAaAG	0.15	
				C: TCTATTACTCTAGGGTCAGATAGAAATTC	0.15	
A.I.8	A.I.9	1453599	C/T	A: GCTGCTGCTAGATTAGCTATgCT	0.15	60
				D: ggggcggggcggggcGCTGCTGCTAGATTAGCTATcCC	0.15	
				C: TCAAGCAATCAACAATAATTTTACTAT	0.15	
A.I.8	A.I.10	797599	T/G	A: GATCAATTGGTGGTGTTcCG	0.80	60
				D: ggggcggggcggggcGTGATCAATTGGTGGTGTTtCT	0.20	
				C: AACGTTTTATCCTCTTGAATATCAACTAT	0.20	
A.I.8	A.I.11	1278606	G/A	A: AAGGAACAAAAAACATCATCATTgCT	0.20	60
				D: ggggcggggcggggcAAAAGGAACAAAAAACATCATCATTaCC	0.20	
				C: TCATACTAACAACGGCTATTCAGGGA	0.20	
A.I.3	NA	1233898	T/G	A: GCTTGACAATATTAGCTTATAAAACTATAgTG	0.15	60
				D: ggggcggggcggggcGCTTGACAATATTAGCTTATAAAACTATAaTT	0.15	
				C: TTTTTTCCATATTTCTGTAAAAAATATACTATTATG	0.15	
A.I.3	A.I.4	830715§§	T/C	A: GTTAAGTCGGTAAGTATCGACAAaTC	0.60	60
				D: ggggcggggcggggcGTTAAGTCGGTAAGTATCGACAAgTT	0.20	
				C: CAAATCTTCTAGTATCTCTTTATCTTCAG	0.20	
A.I.3	A.I.5	113671	G/A	A: cgggcgggcgggcgggGCTTGAGTTTATTTTTTGTTTAATGTgTA	0.20	60
				D: GCTTGAGTTTATTTTTTGTTTAATGTaTG	0.20	
				C: GGACAAAACTGTGGACGTTAAGAA	0.20	
A.I.3	A.I.6	580153	G/A	A: cgggcgggcgggcgggTATAATGGTAACTCATGATCAAGAAcAA	0.20	60
				D: TTATAATGGTAACTCATGATCAAGAAaAG	0.20	
				C: ATCTGTCATGATACCAATTCTTGTCG	0.20	

*Melt-MAMA, melt–mismatch amplification mutation assay; SNP, single nucleotide polymporphism; canSNP, canonical SNP; con, concentration, μmol/L; temp, annealing temperature, °C; NA, not applicable; der, derived SNP state; anc, ancestral SNP state; D, derived allele primer; A, ancestral allele primer; C, common primer. †Genomic position in reference A.I SchuS4 strain (GenBank accession no. NC_006570). ‡SNP states are listed according to their orientation in the SCHU S4 reference genome (GenBank accession no. AJ749949.2). §Melt-MAMA primer sequences; primer tails and antepenultimate mismatch bases are in lower case. ¶Final concentration of each primer in Melt-MAMA genotyping assays. #Assay annealing temperature. **Assay designed on the reverse complement. ††SNP from (*6*). ‡‡Assay supplemented with 0.025 U of Platinum Taq DNA polymerase (Life Technologies, Invitrogen, Frederick, MD, USA). §§SNP from (*7*).

**Figure 2:**
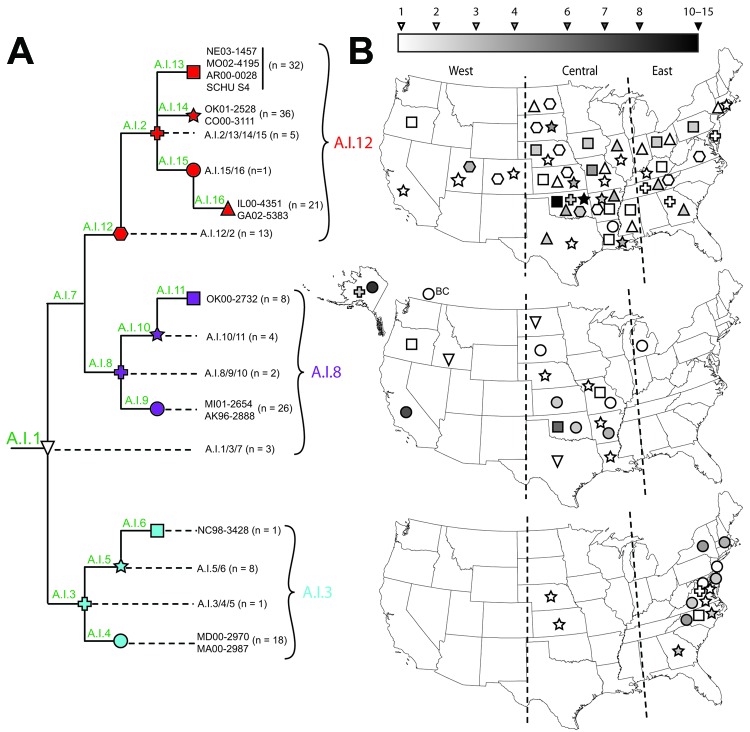
Geographic alignment of 179 geographically diverse *Francisella tularensis* subsp. *tularensis* A.I strains, by subgroup, United States. A) Canonical single-nucleotide polymorphism (canSNP) topology of 15 intervening and terminal subpopulations defined by screening of 16 canSNPs. Colors indicate major subgroups within A.I: red, A.I.12; purple, A.I.8; blue, A.I.3. Subpopulations are indicated by symbols; n values indicate number of strains assigned to each subpopulation. B) Geographic distribution of strains from the 15 subpopulations, shown by corresponding symbols as in panel A and aligned by subgroup (top, A.I.12; middle, A.I.8; bottom, A.I.3). Vertical lines indicate boundaries of the 3 regions: western, central, and eastern. Subgroups are mapped on the basis of geographic origin at the state level. Gradients correspond to number of strains associated with each symbol (i.e., darker symbols indicate a higher number of strains). The basal A.I.1/3/7 subgroup (inverted triangle) cannot be meaningfully assigned to 1 of the 3 main subgroups; thus, this subgroup is arbitrarily represented on the A.I.8 map. BC, British Columbia, Canada.

Each subgroup exhibited complex yet distinct phylogeographic patterns (Figure 2, panel B). Group A.I.12 strains, assigned to 6 subpopulations (Figure 2, panel A), were isolated throughout the United States: all 6 subpopulations were found in the central region, 3 in the western region, and 5 in the eastern region (Figure 2, panel B, top). Group A.I.8 strains, assigned to 4 subpopulations, were found in the central (3 subpopulations) and western (including Alaska and British Columbia; 3 subpopulations) regions, but only 1 strain was isolated in the eastern region (Figure 2, panel B, middle). For group A.I.3 strains, assigned to 4 subpopulations, distribution differed dramatically from the other subgroups; most strains and all 4 subpopulations occurred in the eastern region and just 1 subpopulation in the central region but none in the western region (Figure 2, panel B, bottom).

## Conclusions

The occurrence of the A.I.3 subgroup in the eastern United States could be a recent or ancient event. The subgroup may have been introduced more recently from the central region to a naive niche in the eastern region through importation of rabbits (*Sylvilagus floridanus*) as recently as the 1920s (*3*); before 1937, tularemia was nearly nonexistent in the eastern region (*15*). If the introduction is recent, the current lack of A.I.3 strains in the central United States could be the result of a selective sweep that nearly eliminated this subgroup from its geographic origin. However, most strains and genetic diversity (i.e., subpopulations) within the A.I.3 subgroup are found in the eastern United States, which may reflect a more ancient history in this region involving early introduction and establishment of this subgroup east of the Appalachian Mountains, with only recent spread to the central region.

If we assume that the greatest genetic diversity in a phylogenetic context implies ancient origins, our findings suggest that the central United States is the likely geographic origin of a common ancestor to *F. tularensis* subsp. *tularensis* subgroups A.I.12 and A.I.8 and, perhaps, the A.I group as a whole. The large geographic range of the A.I.12 subgroup and the phylogenetic pattern of a long branch leading to a polytomy with genetic homogeneity point to a possible adaptive advantage for this subgroup. This advantage may be related to difference in virulence among A.I strains, as suggested by previous testing in mice of 2 A.I.12 strains that exhibited lower virulence than that of 2 A.I.3 strains (*11*). Further research is needed to determine whether the genomic differences that define this subgroup are associated with known *F. tularensis* virulence determinants.

Technical Appendix *Francisella tularensis* whole-genome sequencing data generation, assembly, and analysis.
