# Supplementary material for: Francisella tularensis subsp. tularensis Group A.I, United States
Source: Emerg Infect Dis. 2014 May;20(5):861–5. doi: 10.3201/eid2005.131559 (PMC4012810; doi:10.3201/eid2005.131559)
Supplement: Technical Appendix — Francisella tularensis whole-genome sequencing data generation, assembly, and analysis. [file 13-1559-Techapp-s1.pdf]

Article DOI: http://dx.doi.org/10.3201/eid2005.131559

# *Francisella tularensis* subsp. *tularensis* Group A.I, United States

## Technical Appendix

### Genome sequencing and assembly

Sequencing of the 13 *F. tularensis* genomes was performed using an Illumina GA IIx instrument (Illumina Inc., San Diego, CA, USA) (100-bp pair-end reads) at the Translational Genomics Research Institute (TGen; Flagstaff, Arizona, USA) and the sequences were assembled using Abyss v1.3.3 (*1*). To ensure high-quality data, the genome sequences were filtered to minimize uncertain sequence positions in the phylogenetic analysis. After assembly, sequence reads were re-mapped to their corresponding genome sequence using bowtie2 v2.0.0 (*2*) and subsequent SNP-calling by samtool mpileup (*3*) and VarScan v2.3.2 (*4*) using default parameters except p-value=0.9. Using the SNP information, positions for nucleotides that were supported by <90% of the aligned reads were replaced by the nucleotide symbol “N.” After filtering, a multiple genome alignment was calculated by the progressive Mauve algorithm (*5*) using the 13 filtered genome sequences and two public *F. tularensis* genome sequences. These were strain SCHU S4 (acc. AJ749949.2), representing the A.I clade, and strain WY96-3418 (acc. CP000608.1), representing clade A.II and also serving as an outgroup for the phylogenetic analyses. A second filter was then applied to remove all positions within 30-bp of gaps (“-“) or uncertain positions (“n”) to minimize potential misalignment errors. One SNP found to be incorrectly called due to inaccurate mapping in a repetitive region was manually excluded from the alignment.

### Whole genome phylogeny

Based on the filtered and aligned genome data, a Neighbor-Joining tree was inferred using MEGA5 software 2 (Figure 1 in article main text) with gaps/missing data treated as complete deletions.

The naming of the branch leading up to major groups separated by deeply rooted splits was based on SNP nomenclature: A.I.12, A.I.8, and A.I.3 (Figure 1 in article main text). All short read archives were submitted to SRA (NCBI BioProject Accessions: PRJNA187553, PRJNA187555, PRJNA187556, PRJNA187557, PRJNA187558, PRJNA187559, PRJNA187562, PRJNA187563, PRJNA187564, PRJNA187565, PRJNA187567, PRJNA187568, PRJNA187569).

## Single Nucleotide Polymorphism (SNP) identification for the development of new canonical SNP assays

SNPs were identified by mapping paired-end reads to a high quality reference genome (*F. tularensis* SCHU S4, acc. AJ749949.2) (*6*) using BWA short read alignment software (*7*) followed by SNP-calling using samtool pileup (*3*) and VarScan v2.2 (min-var-freq 0.9, min-reads 5 and min-coverage 20) (*4*). Finally, SNPs defining the three clades A.I.12, A.I.8, and A.I.3 were confirmed using an in-house Perl script based on their presence in a multiple alignment of de novo assembled genome sequences. From this information 16 canonical SNP (canSNP) assays were created as previously described (*8*).

### References


1. Simpson JT, Wong K, Jackman SD, Schein JE, Jones SJ, Birol I. ABySS: a parallel assembler for short read sequence data. Genome Res. 2009;19:1117–23. PubMed http://dx.doi.org/10.1101/gr.089532.108

2. Langmead B, Salzberg SL. Fast gapped-read alignment with Bowtie 2. Nat Methods. 2012;9:357–9. PubMed http://dx.doi.org/10.1038/nmeth.1923

3. Li H, Handsaker B, Wysoker A, Fennell T, Ruan J, Homer N, et al. The Sequence Alignment/Map format and SAMtools. Bioinformatics. 2009;25:2078–9. PubMed http://dx.doi.org/10.1093/bioinformatics/btp352

4. Koboldt DC, Chen K, Wylie T, Larson DE, McLellan MD, Mardis ER, et al. VarScan: variant detection in massively parallel sequencing of individual and pooled samples. Bioinformatics. 2009;25:2283–5. PubMed http://dx.doi.org/10.1093/bioinformatics/btp373

5. Darling AC, Mau B, Blattner FR, Perna NT. Mauve: multiple alignment of conserved genomic sequence with rearrangements. Genome Res. 2004;14:1394–403. PubMed http://dx.doi.org/10.1101/gr.2289704

6. Larsson P, Oyston PC, Chain P, Chu MC, Duffield M, Fuxelius HH, et al. The complete genome sequence of *Francisella tularensis*, the causative agent of tularemia. Nat Genet. 2005;37:153–9. PubMed http://dx.doi.org/10.1038/ng1499

7. Li H, Durbin R. Fast and accurate short read alignment with Burrows-Wheeler transform. Bioinformatics. 2009;25:1754–60. PubMed http://dx.doi.org/10.1093/bioinformatics/btp324

8. Birdsell DN, Pearson T, Price EP, Hornstra HM, Nera RD, Stone N, et al. Melt analysis of mismatch amplification mutation assays (Melt-MAMA): a functional study of a cost-effective SNP genotyping assay in bacterial models. PLoS ONE. 2012;7:e32866. PubMed http://dx.doi.org/10.1371/journal.pone.0032866
